# Improvement Efficacy of Influenza Nanovaccine in Combination with Hemokinin-1 Molecular Adjuvant

**Published:** 2018

**Authors:** Atefeh Dehghan, Shahla Shahsavandi, Leila Jabalameli

**Affiliations:** 1. Department of Microbiology, Karaj Branch, Islamic Azad University, Karaj, Iran; 2. Razi Vaccine and Serum Research Institute, Agricultural Research Education and Extension Organization, Karaj, Iran

**Keywords:** Chitosan, Immunization, Influenza vaccines, Influenza virus, Nanoparticles

## Abstract

**Background:**

H9N2 avian influenza viruses have the potential to become the next human pandemic threat and next generation vaccine technologies are needed. Current studies introduce nanoparticles as a proper vaccine delivery vehicle for induction of protective immunity. In this study, the efficacy of chitosan nanoparticle-based H9N2 influenza vaccine with and without hemokinin-1 (HK-1) as a molecular adjuvant to induce protective immunity against the virus was examined.

**Methods:**

The H9N2 antigen was prepared in MDCK cells and inactivated with formalin. The inactivated antigen alone and in combination with HK-1 was encapsulated into chitosan nanoparticles. Groups of BALB/c mice received chitosan nanoparticle-based H9N2 antigen alone or in combination with HK-1 in a prime/boost platform *via* eye drop method. To evaluate the efficacy of the adjuvanted-nanovaccine candidate, systemic antibody responses were compared among the groups of animals.

**Results:**

Serological analysis indicated that mice receiving the HK-1/H9N2 nanoparticles formulation induced higher antibody titers that were sustained until the end of experiment. However, in the immunized mice, influenza specific antibody titers were comparable to that in the animals which were immunized either with inactivated antigen alone or the H9N2 nanoparticles without HK-1 adjuvant.

**Conclusion:**

The data demonstrate the synergy between HK-1 as an adjuvant and chitosan nanoparticles as a delivery antigen/adjuvant carrier in the improvement of influenza immune responses.

## Introduction

Influenza is a worldwide contagious respiratory disease of human, mammals and birds. For decades, chemoprophylaxis and vaccination have been used against influenza infection. Current influenza vaccination program uses either attenuated vaccines or inactivated vaccines to induce protective immunity. Despite the availability of influenza vaccines since the 1930s, direct transmission of avian virus subtypes to human and emergence of new antigenic variants made the efficacy of these vaccines ambiguous. Candidate H9N2 subtype as a potential next pandemic agent has raised a concern about new controlling strategies 1–4.

As a primary idea, development and applications of recombinant proteins and DNA vaccines increased attention. Several studies revealed that vaccines based on the virus-coding DNA vaccines including Hemagglutinin (HA), neucleoprotein, matrix, and neuraminidase antigens are able to increase the level of CD8+ Cytotoxic T Lymphocyte (CTL) and generate antigen-specific immune responses 5–9. These vaccines as well as traditionally inactivated vaccine fail to induce protective immunity when administered without adjuvant because of their poor immunogenic features. Thus, to enhance immune response, influenza antigen is formulated commonly with one of the licensed adjuvants such as aluminum salts, oil-in-water emulsions, and liposomes 10–12.

To induce stronger responses as well as CTL-mediated immunity, molecular adjuvants are also characterized. Compared with the current adjuvants, they act better by enhanced antigen persistence, increased antigen uptake, and recruitment of Antigen Presenting Cells (APCs) 13–17. Moreover, the inactivated vaccines usually administered *via* intramuscular injection may fail to reach the APCs and induction of immune responses. To overcome the difficulty, antigen delivery system is considered as one crucial mechanism in targeting the APCs for increasing the efficacy of vaccines. Currently, many antigen and adjuvant delivery carriers can facilitate the induction of immunity 18–20.

Protection against influenza virus infection greatly depends on both humoral and mucosal immune responses to a major surface glycoprotein, HA 21. Secretory IgA antibodies are the main mediator of upper respiratory tract adaptive mucosal immunity against the influenza. Induction of the immunity is thought to be improved by mucosal delivered vaccine due to a correlation between the amount of IgA antibodies and the efficacy of protection against the infection 18,19. Therefore, a potent influenza vaccine should induce and elevate the antibodies in the host respiratory tract when administrated intranasal. The protection rate is dependent on antigen delivery system that can effectively elicit immune responses.

Nanoparticles are served recently for mucosal antigen delivery system for a wide range of vaccines. The uptake of these particles by APCs can be activated and it can trigger the proliferation of B-cells resulting in class switching of antigen specific plasma cells to produce IgA 20. Among all the available nanoparticles, chitosan, a biopolymer of glucosamine residues, has increased the attention for mucosal delivery system for vaccines. Chitosan is able to release high level of targeted antigen resulting in high long-lived antibody titers, which correlate with protection in case of influenza 21,22.

Hemokinin-1 (HK-1) as a molecular adjuvant can enhance circulating antibody responses when co injected with H9N2 whole inactivated influenza vaccine 16,23. Although the administration route elicits serum antibodies, it does not usually induce mucosal immune responses. Here, the scope of these initial studies was expanded to introduce a vaccine adjuvant/delivery platform capable of enhancing influenza humoral antibodies. The ability of chitosan nanoparticle-based HK-1/influenza vaccine candidate to provide protective immunity against influenza virus was evaluated by consideration of both adjuvant and delivery system.

## Materials and Methods

### Inactivated H9N2 influenza virus antigen preparation

Madin-Darby Canine Kidney (MDCK) cell line (AT CCCCL-34), the permissive cell for influenza virus replication, was maintained in DMEM medium (Sigma, Aldrich) supplemented with 10% FBS, 100 *U/ml* penicillin, and 100 *μg/ml* streptomycin at 37°*C* in a humidified atmosphere of 5% CO_2_. Avian influenza H9N2 virus, A/Chicken/Iran/SS1/1998, was used in this study. Monolayers of the cells at a concentration of 1×10^6^ *cells/ml* were infected with the virus at a multiplicity of infection of 0.1 PFU/cell in the presence of supplemental trypsin. Following adsorption for 1 *hr* at 37°*C*, the inoculum was removed and washed before DMEM was replaced. The cultures were incubated up to 48 *hr* post infection (hpi) and observed by inverted light microscopy for Cytopathic Effect (CPE). The virus was titrated by Hemagglutination Assay (HA) using 0.5% chicken RBCs 24. One HA unit (HAU) is equal to approximately 5 to 6 logs of the virus. The prepared H9N2 antigen was inactivated with a 0.02% final concentration of formalin (Merck) in PBS following incubation for 18 *hr* at 37°*C*.

### HK-1 adjuvant preparation

HK-1 was prepared according to the previous study 16. Briefly, HK-1 sequences from Mus musculus were downloaded from the NCBI database and aligned by the Clustal W algorithm. The coding region was then amplified with the designed primer, HK-1F: 5′-GG ATCCCTTGCCCTGTTTCTCCTGAT-3′ and HK-1R: 5′-CCATGGCTTCCCCATCAGACCGTAAT-3′ carrying the appropriate restriction enzyme sites and cloned into the pcDNA3.1 vector (Invitrogen, USA) between the *Bam*HI and *Nco*I sites, respectively. Plasmid DNA was propagated in *Escherichia coli* (*E. coli*) and purified using EndoFree®Plasmid Mega Kit (Qiagen) according to the manufacturer's instructions. The correct sequence of the cassette within pcDNA3.1 was confirmed by sequencing. Concentration of the purified plasmid was adjusted to 1 *μg/μl*.

### Chitosan nanoparticle-based HK-1/influenza vaccine candidate preparation and characterization

At the first step, chitosan nanoparticles were made by ionic gelation method based on the ionic gelation of chitosan with sodium tripolyphosphate (TPP) anions (Nanozino Co, Iran). In brief, 0.2 *g* of the purified chitosan powder was dissolved in 100 *ml* 0.35% acetic acid solution and pH adjusted to 5.5 by 1 *M* NaOH after overnight incubation. 0.5% TPP was dropped to the chitosan solution under stirring condition for 30 *min* at room temperature. H9N2-loaded nanoparticles were formed by mixing 0.8 *ml* chitosan-TPP at concentration of 0.5 *mg/ml* by 0.1 *ml* H9N2 antigen containing 5 (log_2_) HA. The solution was incubated for 30 *min* at room temperature, then centrifuged at 12,000 *rpm* at 4°*C* for 10 *min*. The amount of free antigen in the solution was determined by HA. The zeta potential (*mV*) value was measured by using Zetasizernano ZS (Malvern, UK). Encapsulation efficiency and loading capacity of the nanoparticles were calculated as follows:

$$\begin{array}{l} \text{Encapsulation}\;\;\text{efficiency}=\text{Total}\;\text{HA}-\text{Free}\;\text{HA}/\text{Total}\;\text{HA}\times 100\%\\ \text{Loading}\;\;\text{capacity}=\text{Total}\;\text{HA}-\text{Free}\;\text{HA}/1\;\mathrm{mg}\;\text{chitosan}\;\text{nanoparticles}\;\text{dry}\;\text{weight} \end{array}$$

By the same method HK-1/H9N2-loaded nanoparticles were made by adding 0.1 *ml* HK-1 (10 *μg/ml*) to the above solution.

Stability of the prepared antigen-loaded and antigen/ adjuvant-loaded nanoparticles during storage at 4°*C* was evaluated by measuring the HA content using Hemagglutination (HA) assay 24. Briefly, 25 *μl* of the reagents were serially diluted 1:2 in PBS in a 96-well plate and incubated for 30 *min* at room temperature with 25 *μl* of chicken red blood cells (1% *v/v*). The endpoint HA titer was defined as the reciprocal of the last highest dilution of reagent th2at caused complete agglutination of red blood cells. HA titers were re-determined once every two weeks to assess the loss of titer over a period of two months.

### Mice immunization trial

Sixty healthy 6–8-week-old female BALB/c mice (Razi Vaccine & Serum Research Institute, Karaj) with 19 to 23 *gr* weight were used. The animals were equally distributed (Table 1) to the control groups (C1 and C2), which received normal saline and inactivated H9N2 antigen, respectively and four treatment groups as follows: groups A1 received chitosan nanoparticle-based H9N2 antigen without HK-1 and group A2 received booster 14 days after the primary vaccination. Group B1received chitosan nanoparticle-based HK-1/H9N2 antigen and group B2 received the same vaccine 14 days after the primary vaccination. 5 *μl* of samples, regarding the groups, were dropped onto both eyes. Before administration, the vaccine was mixed with normal saline. Blood samples were taken from *medial canthus* of the *eye* up to 90 days post inoculation (pi) at 14 days intervals. Influenza virus antigen-specific antibody titers were determined by the endpoint titer dilution method using ELISA assay (IDEXX Influenza AAb test) according to the manufacture’s instruction.

**Table 1.** Experimental groups of BALB/c mice used in the protective potential of HK-1/H9N2 nanovaccine (n=50)

**Group**	**Immunization scheme**
**C1**	Normal saline
**C2**	Inactivated H9N2 antigen
**A1**	Chitosan nanoparticle-based H9N2 antigen without HK-1 (prime)
**A2**	Chitosan nanoparticle-based H9N2 antigen without HK-1 (booster 14 days after prime)
**B1**	Chitosan nanoparticle-based HK-1/H9N2 antigen (prime)
**B2**	Chitosan nanoparticle-based HK-1/H9N2 antigen (booster 14 days after prime)

### Statistical analysis

The statistical analysis was performed with the use of SPSS version 15.0.1. p*-*value of <0.05 was considered statistically significant. The results were expressed as mean±SD for three independent experiments.

## Results

**Figure 1.**
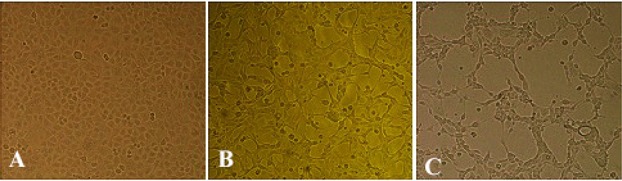
Cytopathogenicity of MDCK cells to H9N2 influenza virus infection: A) mock, and at 24, B) and 48, C) hours post infection (100×).

**Figure 2.**
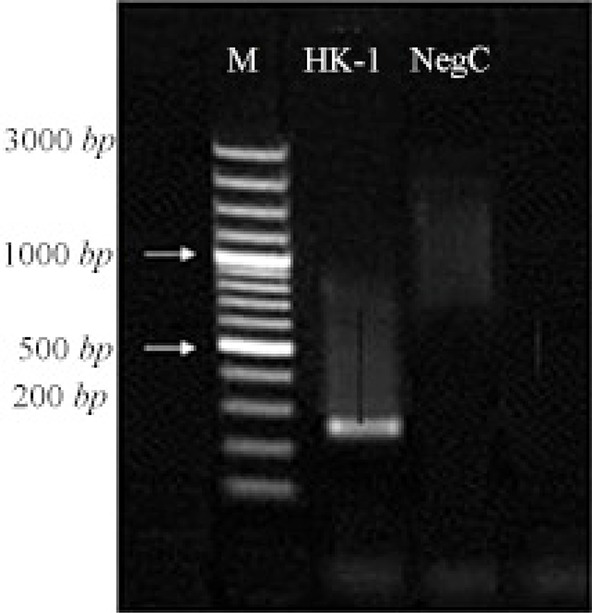
Amplification of HK-1 by specific primers in PCR: M 1 *kb* DNA marker, lane 1 HK-1, lane 2 negative control.

The H9N2 virus was well replicated in MDCK cells in the presence of supplemental trypsin. The infected cell suspension was harvested following observation of CPE at 48 hpi (Figure 1). Virus HA titer was evaluated and found to be 3.70. The influenza virus antigen was further concentrated and formalin-inactivated. The coding region of HK-1 gene was amplified at 231 *bp* and electrophoresed on 1% agarose gel with the specific primers (Figure 2).

Chitosan microparticles were formed by ionic cross-linking between the positive charges of the amino groups of chitosan and the negative charges of TTP ions. Characteristics of the generated chitosan nanoparticle-based HK-1/H9N2 are shown in table 2. The larger size of the loaded nanoparticles than the chitosan-TPP may be due to the molecular weight of H9N2 virus. The zeta potential of the blank chitosan micro-particles was +43.6, which displayed good protein adsorption. The addition of H9N2 inactivated antigen and HK-1 resulted in minor increase in the zeta potential due to the load of positively charged virus surface glycoprotein. Encapsulation efficiency of HK-1/H9N2 on chitosan nanoparticles was estimated 98.43% indicated that the antigen was sufficiently encapsulated. Stability of each nanoparticle formulations was assayed by maintaining the tested vials at 4°*C* for two months. The chitosan nanoparticle-based HK-1/H9N2 and chitosan nanoparticle-based H9N2 retained hemagglutinating activity up to the end of experiment compared to the inactivated H9N2 antigen. The HA titers (log_2_) of the nanoparticles remained 7 when tested after one month post incubation at 4°*C*, whereas the titer of H9N2 antigen dropped by 3log_2_. At the end of experiment, no change on HA values of the nanoparticles was observed; while H9N2 antigen lost HA activity.

**Table 2.** The characteristics loaded H9N2 influenza antigen and hemokinin-1 as adjuvant on chitosan nanoparticles

**Particle size (*nm*)**		**Zeta potential (*mV*)**	
**Blank**	**Ag/Adj loaded-nanoparticles**	**Blank**	**Ag/Adj loaded-nanoparticles**
254±21.7	338±22.6	43.6±1.2	47.7±0.9

**Figure 3.**
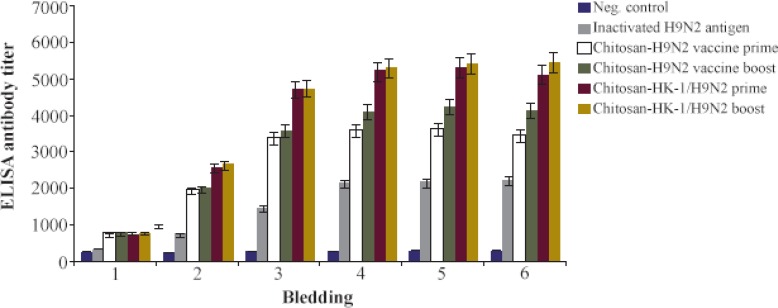
Efficacy of H9N2 influenza nanovaccine adjuvanted with HK-1 in mice. The ELISA antibody titer results show that the candidate vaccine could induce efficacious humoral immune responses against influenza virus (p<0.05).

Efficacy of the generated chitosan nanoparticle-based HK-1/H9N2 vaccine candidate was evaluated in mice. The mice in vaccinated and control groups gained weight from 81.3 to 96.5%. All animals survived after vaccination indicating that the H9N2 antigen and the nanovaccine formulations were safe. The potential of HK-1 and chitosan nanoparticles was evaluated regarding whether they can enhance specific antibody production in response to H9N2 influenza vaccination. Chitosan nanoparticle-based H9N2 antigens with or without HK-1 induced humoral immunity in mice against H9N2 antigen, while loading H9N2 antigen and HK-1 onto the nanoparticles coated with chitosan carrier developed an improved immune response (Figure 3). The antibody production level was significantly higher (*p*<0.05) in groups B1 and B2 indicating chitosan nanoparticles loaded H9N2 antigen with HK-1 adjuvant induced immune responses against influenza; however, group B2 with twice vaccination showed a marginally higher antibody level. These results show that chitosan nanoparticle enhances immune responses in influenza immunization. Increased antibody levels in mice received chitosan nanoparticle-based HK-1/H9N2 antigen revealed that HK-1 adjuvant formulation conferred superior protection over the adjuvant-free formulation.

## Discussion

Avian influenza is a significant global concern because of its potential to become the pandemic threat. New strategies should be taken into account in order to control the infection in human and bird populations. Studies with nanoparticle-based vaccine candidates evidenced their ability to stimulate mucosal as well as systemic immunity 18,19,21. In our study, it was shown that the chitosan as nano-antigen/adjuvant delivery system is effective to encapsulate viral antigen and induce immune response against influenza. The immunogenicity of the inactivated H9N2 antigen improved when HK-1 was used as an adjuvant.

Most current inactivated vaccines are administrated by intramuscular, subcutaneous, or intradermal injection. Although humoral immunity often provides good protection against influenza, mucosal vaccines may be necessary for induction of long-term protection 25. A variety of polycationic polymers and their derivatives were explored to stimulate immune system but they have not been an unqualified success, suggesting that both adjuvant and delivery system may need reconsideration. Some researches focus on the ability of chitosan nanoparticles as a novel adjuvant to induce specific antibody titer against influenza antigens. Sawaengsak *et al* 26 found that the chitosan nanoparticle encapsulated HA-split vaccine reduced markedly the influenza morbidity and also conferred 100% protective rate to the vaccinated mice against lethal influenza virus challenge. Khalili *et al* 27 also showed that the nanoparticles significantly enhanced the immunogenicity of inactivated H9N2 influenza virus in chickens in comparison to ISA70, an oil adjuvant. Similar results on chitosan nanoparticle advantages have been reported by other researchers 28–30. Taken together, loading influenza vaccines on chitosan nanoparticle and its derivatives can induce efficient immune responses against the viruses indicating the adjuvant property of these polysaccharides.

According to comparative analysis, it was found that H9N2 antigen plus HK-1 encapsulated into chitosan nanoparticles showed significant higher levels of influenza specific antibodies. This improvement can be due to formulation of the inactivated antigen with HK-1. It seems that the adjuvant is more effective in inducing antigen response and stimulates B cells responses than chitosan. Previous study showed that HK-1 is a potent adjuvant for the immunization of inactivated influenza virus vaccine and intramuscularly administration of the adjuvanted vaccine significantly enhanced production of specific antibodies in systemic immunity 16. HK-1 is expressed by mature dendritic cells and can promote immune cell survival and activation. The cells play key roles in the induction of antigen-specific effector T helper responses 31–33. There is no doubt on potential adjuvant properties of HK-1 in induction of type-1 CD4+ T helper as well as CD8+ CTL responses resulted in enhancing humoral and memory responses.

To provide an advantageous approach for eliciting higher immunogenicity upon vaccination, mucosal delivery was considered for the inactivated antigen to trigger mucosal immunity as well as systemic immune responses. Therefore, chitosan nanoparticles were applied as carrier during influenza antigen and HK-1 adjuvant *via* eye drop vaccination because the eye mucosa shares common immunologic features with nasal counterpart.

In mucosa delivery system, antigen/adjuvant can be taken by M-cell in the mucosal epithelial and processed by APC to activate B-cells 18. It has been shown that chitosan nanoparticles can stimulate and enhance humoral antibody titers by up regulation of CD69 expression on B cells and CD4^+^T lymphocytes 34. CD69 is the early activation marker for B cells expressed on the surface of activated leukocytes and involved in lymphocyte migration and cytokine secretion. This activation is normally accompanied by the rapid class-switching of the activated lymphocytes into IgA producing plasma cells which subsequently resulted in elevation of IgG and enhancing IgA antibody responses.

CD69 marker is critical for the generation and maintenance of professional memory T-helper lymphocytes, which can efficiently facilitate humoral immunity 34,35. The ability of chitosan for mucosal adhesion and antigen trans-mucosal presentation, and its role on induction of immunity indicated that the nanoparticle is an effective carrier with potential adjuvant property 21,22,36. Here, it was found that chitosan appeared to have a role in enhancing the systemic antibody responses in the mice groups immunized either with chitosan-loaded H9N2 antigen and HK-1 formulations or antigen only. This evidence supports chitosan as a robust immune potentiator for mucosal vaccination. The loaded chitosan nanoparticles easily release H9N2 antigen through the cell membranes and enhance antigen uptake by mucosal lymphoid tissues which improves the mucosal-based immunological responses. The improvement is dependent on the reversible interaction of chitosan with antigen/adjuvant that allows antigen uptake and enhances antigenic trans-mucosal delivery. The size, high positive charge, and hydrophobicity of chitosan are also added to the reversible binding to make it a suitable delivery carrier 37.

In this study, an attempt was made to assess whether a booster vaccination could improve antibody responses against influenza. Following the primary immunization, antibody titers increased in mice either received chitosan nanoparticle-based HK-1/H9N2 or chitosan nanoparticle-based H9N2 with a comparable level in control. In both treated groups, the booster vaccination led to a rise in influenza specific antibody, which was not statistically significant between the prime and boost in each group. These data suggest that a prime immunization regimen conferred protection; however, formulation of influenza nanoparticle with HK-1 offers a potential efficacy advantage.

## Conclusion

It was proposed that eye drop vaccination of HK-1/H9N2 nanoparticles is an effective and safe tool to induce protective immunity against influenza virus infection. Other advantages of the vaccine candidate are stability at 4°*C* which does not require low temperature storage, good tolerability, non-toxic and easy administration. More studies need to be carried out to optimize the adjuvanted-nanovaccine candidate for use in commercial applications.
